# Role of Fibroblasts in Chronic Inflammatory Signalling in Chronic Rhinosinusitis with Nasal Polyps—A Systematic Review

**DOI:** 10.3390/jcm12093280

**Published:** 2023-05-04

**Authors:** José Palacios-García, Cristina Porras-González, Ramón Moreno-Luna, Juan Maza-Solano, Juan Polo-Padillo, José Luis Muñoz-Bravo, Serafín Sánchez-Gómez

**Affiliations:** 1 Department of Otorhinolaryngology and Head and Neck Surgery, University Hospital Virgen Macarena, Doctor Fedriani 3, 41009 Seville, Spain; 2 Institute of Biomedicine of Seville (IBiS), Campus Hospital Universitario Virgen del Rocío, Avda. Manuel Siurot s/n, 41013 Sevilla, Spain; 3 Department of Medical Biochemistry, Molecular Biology and Immunology, School of Medicine, Virgen Macarena University Hospital, University of Seville, Doctor Fedriani 3, 41009 Seville, Spain; 4 Department of Preventive Medicine and Public Health, University Hospital Virgen Macarena, Doctor Fedriani 3, 41009 Seville, Spain; 5 Clinical Analysis Service, General University Hospital of Elche, Foundation for the Promotion of Health and Biomedical Research in the Valencia Region (FISABIO), Av. De Catalunya 21, 46020 Valencia, Spain

**Keywords:** nasal polyps, chronic rhinosinusitis, fibroblasts, nasal-polyp-derived fibroblasts, cytokines, inflammatory cytokines

## Abstract

Chronic rhinosinusitis with nasal polyps (CRSwNP) is an inflammatory disease of the nose and paranasal sinuses characterized by the presence of nasal polyps. The symptoms produced by the presence of nasal polyps such as nasal obstruction, nasal discharge, facial pain, headache, and loss of smell cause a worsening in the quality of life of patients. The source of the nasal polyps remains unclear, although it seems to be due to a chronic inflammation process in the sinonasal mucosa. Fibroblasts, the main cells in connective tissue, are intimately involved in the inflammation processes of various diseases; to this end, we carried out a systematic review to evaluate their inflammatory role in nasal polyps. Thus, we evaluated the main cytokines produced by nasal polyp-derived fibroblasts (NPDF) to assess their involvement in the production of nasal polyps and their involvement in different inflammatory pathways. The results of the review highlight the inflammatory role of NPDF through the secretion of various cytokines involved in the T1, T2, and T3 inflammatory pathways, as well as the ability of NPDF to be stimulated by a multitude of substances. With these findings, the fibroblast is positioned as a new potential therapeutic target in the treatment of CRSwNP.

## 1. Introduction

Chronic rhinosinusitis with nasal polyps (CRSwNP) is a subtype of chronic rhinosinusitis (CRS) that affects 18–20% of all patients with CRS and 1 to 4% of the world population [1,2]. Patients suffering from this condition are mainly adult males with a 2:1 preponderance, the disease being rare during childhood, where it is usually found in association with cystic fibrosis [3]. The quality of life of patients suffering from CRSwNP is greatly impaired. It is essentially due to the discomfort caused by the main symptoms, such as difficulty breathing and constant runny nose, but also due to the indirect effects of symptoms such as loss of smell, taste, headaches, changes in sleep patterns, daytime sleepiness, as well as decreased intellectual capacity [2].

The main phenotypic characteristic of these patients is the growth of nasal polyps generally bilateral at the level of the middle meatus, although they can also be found in the paranasal sinuses [4]. Macroscopically, nasal polyps are bright yellowish-grey, shiny, translucent masses composed of gelatinous inflammatory material, and they have a benign behaviour. Histologically, they are described as an oedematous myxoid stroma, infiltrated mainly by eosinophils covered by a respiratory epithelium that frequently presents hyperplasia or squamous metaplasia [5]. Therefore, in the inflammatory infiltrate of nasal polyps, fibroblasts appear to be one of the most abundant cells in polypoid tissues (47%) followed by eosinophils (20%) [6], and other inflammatory cell types, such as neutrophils, mast cells, lymphocytes, dendritic cells, plasma cells, and ILC2s [7,8,9,10].

The cause of the development of these polypoid lesions remains unclear, although advances in the understanding of the pathophysiology of the disease suggest that they are the product of a chronic inflammatory mechanism. There are several theories as to why this sustained inflammation would take place, such as an alteration of the mucosal barrier, the presence of microorganisms (such as bacteria, fungi, or viruses), the production of superantigens, or the presence of allergens [11]. Until recently, the inflammatory profile leading to CRSwNP was believed to be mediated almost exclusively by a Th2 response. Nevertheless, recent studies have shown that the inflammatory response is more complex and heterogeneous, with the involvement of three different inflammatory endotypes T1, T2, and T3 [2]. These endotypes are characterized by the secretion of various cytokines depending on the participation of Th1, Th2, and Th17 helper cells. Therefore, IFN-γ secretion is predominant in the T1 endotype, while the T2 endotype cytokines are characterized by IL-4, IL-5, and IL-13 cytokines, as well as eosinophilia, and the T3 endotype is associated with IL-17cytokine expression and neutrophilia [12]. Currently, many of these cytokines are used as therapeutic targets against certain CRSwNPs, such as IL-4 with dupilumab or IL-5 with mepolizumab.

Fibroblasts are the main cell type found in connective tissue and are mainly known for their production of the extracellular matrix (ECM). However, they are not only involved in the production of the ECM but are also intimately involved in inflammation due to their capacity to secrete cytokines, adipokines, and growth factors [13]. Thus, these mesenchymal cells appear to be directly involved in the innate host defence and have been linked to various inflammatory diseases such as rheumatoid arthritis [14], inflammatory bowel disease [15], lung inflammation [16], or nasal polyposis [17]. However, fibroblasts differ according to the anatomical site, varying their function, proliferation, and production of substances according to the requirements of the tissue environment [18]. Thus, we evaluated the main cytokines produced by nasal-polyp-derived fibroblasts (NPDF) to assess their involvement in the production of nasal polyps. This systematic review was designed with the aim of investigating the main cytokines secreted by NPDF, and their involvement in different inflammatory pathways.

## 2. Methods

A systematic review of the literature was carried out to study the main cytokines produced by nasal polyp fibroblasts.

### 2.1. Research Questions and Eligibility Criteria

With this systematic review, we aimed to answer the following research question: What are the cytokines involved in the inflammatory response produced by fibroblasts in patients with nasal polyps? This question was defined in terms of the PICO format. The criteria for the inclusion/exclusion of studies are shown in Table 1. The types of studies included were clinical trials, prospective, and retrospective studies published in peer-reviewed journals. Clinical cases and articles dating before 2000 were excluded. We also excluded articles that evaluated the inhibition of a cytokine or inflammatory pathway. The search was last updated in May 2022. Only studies published in English language were included.

### 2.2. Search Strategy

We followed the Preferred Reporting Items for Systematic Reviews and Meta- Analysis (PRISMA) [19], and the search was carried out in the following databases: Pubmed (Medline), SCOPUS, EMBASE, Web of Science, and the Cochrane Library. The search terms used were as index terms or free-text words and the complete strategy was as follows: ((“nose polyp” OR “nasal cavity polyp” OR “nasal polyp” OR “nasal polyposis” OR “nasal polyps” OR “nose polyp” OR “nose polyposis” OR “polyp of the nasal cavity” OR “polyp of the nose” OR “polyp, nose” OR “polyposis nasi” OR “polyposis of the nose” OR “chronic rhinosinusitis with nasal polyp”) AND (“fibroblast” OR “fibroblast cell” OR “fibroblasts” OR “primary fibroblast” OR “primary fibroblasts”) AND (“inflammation” OR “inflammation reaction” OR “inflammation response” OR “inflammatory condition” OR “inflammatory reaction” OR “inflammatory response” OR “reaction, inflammation” OR “response, inflammatory” OR “cytokine” OR “cytokines” OR “interleukin”)).

### 2.3. Data Collection

Two reviewers (JPG, SSG) independently screened the references based on the title and abstract followed by eligibility at the full-text level. In case of disagreements between reviewers when reviewing abstracts, articles were included in the full-text review phase for a final evaluation. The references of all selected articles were also manually reviewed to identify any potentially omitted publications.

### 2.4. Data Analysis

Results are discussed in narrative and table format.

## 3. Results

### 3.1. Search Results

**Figure 1:**
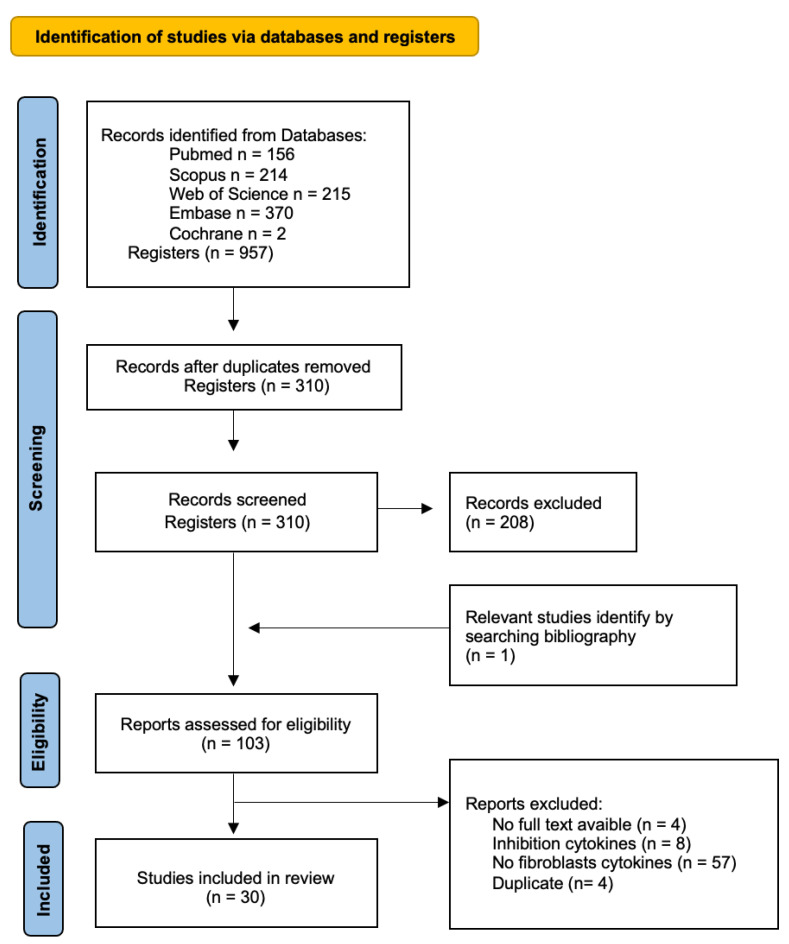
Flow diagram of the identification, screening, and inclusion of studies within the systematic review.

The initial search generated a total of 957 articles: 156 in Pubmed, 214 in Scopus, 215 in Web of Science, 370 in Embase, and 2 in Cochrane. Duplicate references found in different databases were removed. Next, we screened by title and abstract, including 102 articles for the full-text analysis. This analysis included a total of 30 articles to review. The flow chart of the literature search and selection process is shown in Figure 1. The baseline characteristics of the 30 articles included are presented in Table 2.

### 3.2. NPDF Cytokines

**Figure 2:**
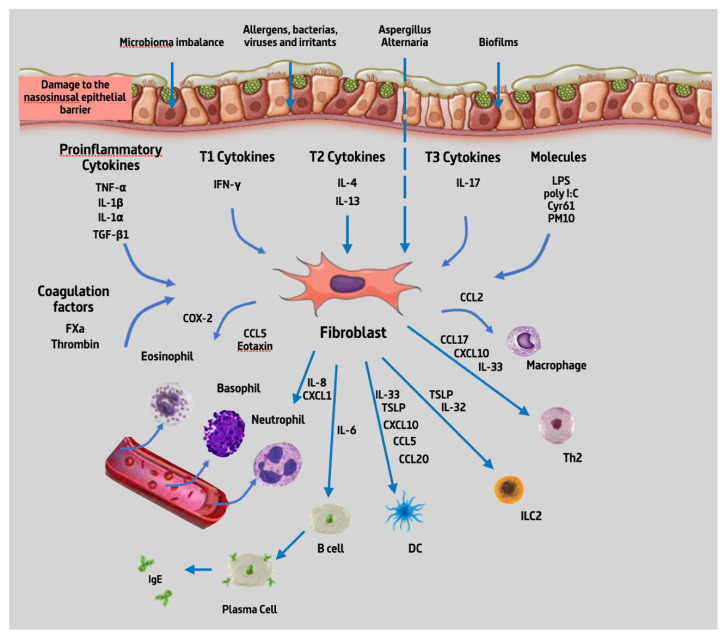
Summary of cytokines produced by NPDF. Abbreviations: TNF-α, tumour necrosis factor alpha; IL-1β, interleukin 1 beta; IL-1⍺, interleukin 1 alpha; TGF- β, transforming growth factor beta; IFN-γ, interferon gamma; IL-4, interleukin 4; IL-13, interleukin 13; IL-17, interleukin 17; LPS, lipopolysaccharide; poly IC, polyinosinic:polycytidylic acid; Cyr61, cysteine-rich angiogenic inducer 61; PM10, particular matter smaller than 10 µm; FXa, coagulation factor Xa; COX-2, cyclooxygenase-2; CCL5, chemokine (C-C motif) ligand 5; IL-8, interleukin 8; CXCL1, C-X-C motif chemokine ligand 1; IL-6, interleukin 6; IL-32, interleukin 32; IL-33, interleukin 33; TSLP, thymic stromal lymphopoietin; CXCL10, C-X-C motif chemokine ligand 10; CCL20, chemokine (C-C motif) ligand 20, CCL17, chemokine (C-C motif) ligand 17; CCL2, chemokine (C-C motif) ligand 2; B cell, B lymphocytes; DC, dendritic cell; ILC2, type 2 innate lymphoid cells; Th2, T helper 2 lymphocytes; IgE, immunoglobulin E.

From the 32 articles reviewed, we found that NPDF could produce 19 different types of cytokines upon stimulation with about 22 different types of substances. Given the high number of cytokines produced by NPDF and substances capable of producing a stimulation, they are summarized in Figure 2 below.

## 4. Discussion

Fibroblasts are present in all tissues and play a fundamental role in processes such as wound healing, ECM remodelling, and the modulation of a tissue-specific immune response [20]. They represent the main type of mesenchymal cells and have the ability to receive signals from the epithelial barrier upon damage. At the same time, fibroblasts can emit signals to activate innate immune cells [21]. In fact, NPDF themselves, as well as other fibroblasts of other tissues, appear to act as sentinels of tissue-specific innate immune responses by producing inflammatory mediators such as cytokines, chemokines, antimicrobial peptides, and growth factors in response to various substances [22]. NPDF actively participate in epithelial remodelling and proliferation, while modulating ECM through a proinflammatory transcriptional programme with resistance to apoptotic signalling, which may explain the maintenance of inflammatory signalling in the production of nasal polyps [23].

### 4.1. Chemokine C-C Motif Ligand 5 (CCL5)

NPDFs are capable of expressing the cytokine CCL5, also known as RANTES, upon stimulation with proinflammatory cytokines such as TNF-⍺, IL-1β, and IL-4 [24,25,26]. CCL5 (RANTES) is a chemoattractant protein of various types of inflammatory cells, such as monocytes, memory T cells, basophils, macrophages, mast cells, and eosinophils [27]. In CRSwNP, RANTES has been proposed to act as a strong attractant of eosinophil infiltration [28]. However, Yoshikufu et al. [26] found no differences in RANTES expression levels in eosinophil-rich nasal polyp versus noneosinophilic nasal polyp, indicating that increased RANTES secretion may not be associated with eosinophilic infiltration in nasal polyps. The transcriptional upregulation of RANTES is mediated by nuclear factor κB (NFκB) following the activation of TLR1/TLR2, TLR3, and NOD1 [29]. These fast intracellularly acting pathways are generally present in the innate response of the immune system generally after contact with pathogen-associated molecular patterns (PAMPs), suggesting that NPDF may be involved in the initial response to epithelial damage.

### 4.2. Eotaxins

Eotaxins are a family of eosinophil recruitment chemokines that carry out their activity through CCR3 receptors [30]. In humans, there are three types of eotaxins: CCL11 (eotaxin-1), CCL24 (eotaxin-2), and CCL26 (eotaxin-3). Terada et al. [31] reported that nasal mucosa fibroblasts were the major source of eotaxins in the nasal mucosa. Nasal fibroblasts are capable of producing at least CCL11 (eotaxin-1) and CCL26 (eotaxin-3) but do not appear capable of secreting CCL24 (eotaxin-2) [32]. NPDF produces CCL11 (eotaxin-1) nonconstitutively upon IL-4 stimulation or costimulation with lipopolysaccharide (LPS) and IL-4 [33,34]. Furthermore, CCL11 (eotaxin-1) appears to be more upregulated in patients with eosinophilic nasal polyps than in those with noneosinophilic polyps following IL-4 stimulation or co-stimulation with TNF-α and IL-4 [26]. On the other hand, Shimizu et al. [35] also verified the production of eotaxin-1 after induction with activated coagulation factors such as thrombin or FXa, which highlights the role of coagulation factors in CRSwNP. Recently, it has been shown that NPDF can produce CCL26 (eotaxin-3) after stimulation with IL-4, IL-13, and leptin [36]. NPDF eotaxin production participates not only in the recruitment of eosinophils, with a subsequent secretion of cytokines, but also in the leakage of plasma into polypoid tissue with the recruitment of other substances such as leptin or coagulation factors, which may favour the development of nasal polyps.

### 4.3. Interleukin-4 (IL-4)

IL-4 is a cytokine that promotes the differentiation of Th2 lymphocytes and B lymphocytes [37]. In patients with CRSwNP, IL-4 is normally elevated and is associated with its involvement in the loss of epithelial integrity by decreasing the expression of occluding epithelial tight junction proteins and zonula occludens [38]. The production of IL-4 by NPDF was demonstrated by Lee et al. [39] after a stimulation of NPDF with particulate matter <10 μm (PM10). This IL-4 production after PM places air pollution as a risk factor for the development of CRSwNP [40,41]. Steinke et al. [34] found that NPDF contained IL-4 receptors (IL-4R) and that their activation caused the upregulation of TGF-1, IL-6, CCL11 (eotaxin-1), and CCL13 (MCP-4). This upregulation occurred in constitutively expressed cytokines, except for CCL11 (eotaxin-1), which did not appear to be constitutively expressed, probably due to the need for eosinophilic regulation by NPDF. This migration of eosinophils can be promoted by a single stimulation, with IL-4 secreting CCL13 (MCP-4) [42], or by combined stimulations of IL-4 with TNF-⍺, LPS, and TFG-β1 [26,33].

However, many other constitutive cytokines are not activated by IL-4 such as IL-6, IL-8, CCL5 (RANTES), CCL13 (MCP-4), and GM-CSF. Furthermore, IL-4 may have an inhibitory role in the secretion of some interleukins by NPDF, such as the inhibition of IL-11 [34]. This inhibition of interleukins, such as IL-11, may be crucial in maintaining the inflammatory state in nasal polyps, as IL-11 has been shown to attenuate inflammation in diseases such as rheumatoid arthritis or inflammatory bowel diseases [43,44].

### 4.4. Interleukin-6 (IL-6) and (IL-8)

IL-6 and IL-8 production is generally increased in CRSwNP [45,46], as well as other inflammatory airway diseases such as asthma and COPD [47,48]. IL6- and IL-8 have as their main function neutrophil chemotaxis. In addition to this shared function, IL-6 is also involved in increasing epithelial cell proliferation [49], while IL-8 is involved in angiogenesis [50]. NPDF are capable of producing these cytokines together or separately after stimulation with various substances. Liu et al. [51] found that the NPDF secretion of IL-6 after stimulation with TNF-⍺ and IL-4 played a role in the pathogenesis of nasal polyps by modulating the immune response and ECM secretion via cycloxygenase-2 (COX-2). Cho et al. [52] confirmed that the COX-2 pathway was important for the production of IL-6 and IL-8 by stimulating NPDF with prostaglandin E2 (PGE2), a metabolite of COX-2, proving that its activation depended on the Akt and the NFκB pathways. The expression of IL-6 and IL-8 by NPDF also occurs after stimulation with cellular products. LPS is capable of producing IL-6 and IL-8 secretion through the PI3K/Akt pathway [53]. Alternaria stimulus, a saprophytic fungus, also produces the NPDF secretion of IL-6 and IL-8 through pattern recognition receptors (PRR), receptors present in the innate immune system for the recognition of microbial molecules [54]. Peripheral blood mononuclear cells (PBMC) stimulated with the house dust mite *Dermatophagoides pteronyssinus* also produce an increase in IL-6 and IL-8 [55].

Shun et al. [56] demonstrated the production of IL-8 and VEGF by NPDF under hypoxic conditions, leading to angiogenesis and the neutrophil infiltration of nasal polyps. This IL-8 production by NPDF can also occur after stimulation with enzymes of the coagulation cascade, such as factor Xa or thrombin, thus indicating that the coagulation system is an inducer of cytokine production in nasal polyps [35] and is probably involved in the accumulation of extracellular fluid required for polyp formation.

### 4.5. Interleukin-13 (IL-13)

IL-13 is a cytokine whose main function is to regulate monocyte and B cell function [37]. In addition to these main functions in patients with CRPScPNS, it has been implicated in the disruption of the epithelial barrier [57], goblet cell hyperplasia [58], and eosinophil recruitment [33]. The expression of IL-13 by NPDF has not been previously reported, but it appears capable of its recognition when expressing the alpha IL-13 receptor (IL-13R⍺) [59]. The IL-13R⍺1 subunit appears to be constitutively expressed, while the IL-13R⍺2 subunit needs to be previously stimulated with TNF-⍺ and/or IL-4 to be upregulated. The synergistic combination of these proinflammatory cytokines (TNF-⍺ and IL-4) induces the upregulation of IL-13R⍺2 but not IL-13R⍺1. IL-13R⍺2 has a high affinity for IL-13 binding and appears to act as a decoy receptor that inhibits STAT6, the canonical IL-13 activation pathway [60,61]. Additionally, IL-13R⍺2 also inhibits the action of IL-4 because this cytokine shares with IL-13 the IL-4 type II receptor for cell signalling [62]. These inhibitory mechanisms may be part of a cellular regulatory feedback in the face of exaggerated inflammatory stimuli by dysregulated IL-4 and IL-13 signalling.

### 4.6. Interleukin-17 (IL-17)

The T3 inflammatory pathway is dominated by Th17 and ILC3 cells, which characteristically produce IL-17A and IL-22 [63,64]. IL-17 is involved in autoimmune inflammation processes and allergic reactions [65]. This signalling pathway mainly produces a neutrophil recruitment, and according to the study by Cheng et al. [66], NPDF, upon stimulation by TNF-α, are able to produce increased levels of IL-17A via the PI3K/Akt pathway, thus indicating that NPDF may participate in some way in the T3 inflammatory pathway by recruiting neutrophils in cases of CRSwNP without eosinophilia. In addition to the ability to secrete IL-17A, this cytokine can also activate NPDF to produce IL-8 and CXCL1. NPDF secretion of these two cytokines after IL-17 stimulation is also correlated with neutrophil chemotaxis, which in turn would lead to an increased IL-17 secretion, accumulating more neutrophils in the nasal mucosa, therefore causing a chronic inflammatory loop [67,68]. Homma et al. [69] confirmed the IL-8 and CXCL1 production after stimulation with IL-17, but they also proved that the stimulation with IL-17 also produced IL-6, IL-9, MCP-1 (CCL2), G-CSF, cytokines involved in the attraction of eosinophils.

The other main interleukin in the T3 pathway is IL-22, which is a fundamental mediator of inflammation after tissue damage, activating the epithelial cells of many organs, including fibroblastic cells [70]. This interleukin has other functions such as mucus production, protective function against pathogens, wound healing, and tissue regeneration that may be deregulated in chronic inflammation [71]. However, in our review, we could not find any study analysing NPDF stimulation with this cytokine or reporting IL-22 production by NPDF.

Although endotyping has proven to be pivotal in the management of chronic inflammation of CRSwNP, the actual clinical presentation may comprise a spectrum with overlapped inflammatory responses. Indeed, Hao et al. [72] found that IL-17A production correlated in turn with IFN-γ production, which highlights the existence of a synergistic effect between the T1 and T3 inflammatory response in the pathogenesis of certain patients with CRSwNP.

### 4.7. Interleukin-32 (IL-32)

IL-32 is a proinflammatory cytokine produced by monocytes, T cells, natural killer cells, dendritic cells, endothelial and epithelial cells, and fibroblasts [73,74]. IL-32 expression is induced by numerous cytokines such as TNF-α, IL-1β, IL-12, and IL-18 [75,76]. Keswani et al. [77] found that IL-32 mRNA was significantly increased in nasal polyp tissue from patients with CRSwNP compared to uncinate tissue from the same patients and healthy controls. Similarly, Soyka et al. [78] also demonstrated that IL-32 mRNA expression levels were significantly increased in nasal polyp compared to control tissues. Cho et al. [79] demonstrated that NPDF could induce IL-32 production when stimulated by LPS, and this effect was mediated by the TLR4/JNK/AKT/CREB pathway.

Interleukin-33 (IL-33) is a cytokine that belongs to the IL-1 superfamily group. It can be expressed by various cell types such as mast cells, macrophages, dendritic cells, fibroblasts, osteoblasts, epithelial, and endothelial cells [80] and induces many immune cells such as ILC2, T helper cells, mast cells, eosinophils, dendritic cells, and basophils [81]. It has been described as being involved in allergic and nonallergic inflammation via the IL-33/ST2 pathway, being considered an alarmin following tissue damage [82]. Recently, Lee et al. [39] described the ability of NPDF to secrete IL-33 after PM10 stimuli, suggesting that NPDF may be one of the first cells to participate in the inflammatory response of the nasal mucosa of patients with nasal polyps.

### 4.8. CXC Motif Chemokine Ligand 10 (CXCL10)

CXCL10, also known as interferon gamma-induced protein 10 (IP-10), is a cytokine secreted in response primarily to IFN-γ by cells such as monocytes, fibroblasts, and endothelial cells [83]. CXCL10 can function as a chemoattractant for T cells, monocytes, NK cells, and dendritic cells, in addition to its role in cell adhesion functions, antitumor activity, angiogenesis, and inhibition of bone marrow colonies [83,84]. Yoshikawa et al. [85] found that CXCL10 expression by NPDF was upregulated by poly IC from CRS patients with asthma and this induced a Th1 cell infiltration into nasal polyp tissues. This activation of the T1 inflammatory pathway by viral immunostimulatory particles appears to be inhibited by bacteria such as S. aureus, which appears to downregulate IP-10 production and prevent Th1 cell recruitment [86]. Recently, Nam et al. [87] pointed to elevated levels of IkappaB kinase (IKK), in particular IKKε, as the trigger for severe eosinophilic inflammation with CXCL10 overproduction.

### 4.9. Chemokine C-C Motif Ligand 2 (CCL2)

CCL2 is a small cytokine that belongs to the CC chemokine family [88]. Its main function is to recruit monocytes/macrophages into tissues; hence, it is also called monocyte chemoattractant protein 1 (MCP1). NPDF can produce CCL2 after the activation of TNF-⍺ mainly through the B-Raf/MEK/ERK pathway, although also with the participation of transcriptional factors such as c-Fos and AP-1 [89,90]. The macrophage-stimulating potential of CCL2 is correlated with COX-2 expression, which is, in turn, associated with the development of polypoid lesions, such as nasal polyps [91] or colonic polyps [92].

### 4.10. Chemokine C-C Motif Ligand 17 (CCL17)

CCL17, also called thymus and activation-regulated chemokine (TARC), is a cytokine belonging to the CC chemokine family and is associated with type 2 immune responses by inducing T cell chemotaxis through its interaction with the CCR4 chemokine receptor [93]. NPDF can produce CCL17 (TARC) through the combined stimulation of IL-4 and LPS [94]. Nonaka et al. [95] demonstrated that NPDF did not induce CCL17 (TARC) production after individual stimulation with TLR ligands. However, a combined stimulation of different TLRs, such as TLR2, 3, 4, 5 ligands, together with IL-4, induced an increase in CCL17 (TARC) secretion. The same author found that the stimulation of NPDF with poly IC, poly IC and TNF-⍺, poly IC and IL-4, or IL-4 and TNF-⍺ induced a significant increase in the expression of TARC. Furthermore, the simultaneous exposure of cells to poly IC, IL-4, and TNF-⍺ induced an even higher increase in the expression of CCL17 (TARC) [96,97]. In view of these findings, IL-4 seems fundamental in the induction of CCL17 (TARC) expression by NPDF and in the regulation of T2 cell chemotaxis.

### 4.11. Chemokine C-C Motif Ligand 20 (CCL20)

Chemokine ligand 20, also known as macrophage inflammatory protein-3 (MIP-3α), is a chemokine that attracts lymphocytes and to a lesser extent neutrophils [98]. It interacts with the CCR6 receptor, a property shared with antimicrobial beta-defensins [99]. It is produced by dendritic cells (DC), monocytes, granulocytes, T cells, B cells, epithelial cells, endothelial cells, and synovial fibroblasts [75,100], and its production is induced by LPS, TNF-⍺, and IFN-γ and deregulated by IL-10 [101]. NPDF are capable of producing CCL20 (MIP-3α) upon stimulation with cytokines such as IL-1β and TNF-α, but also by TLR2, 3, 4, and 5 ligands, suggesting the ability of NPDF to recruit dendritic cells [6]. In addition, IL-17 has also been shown to act synergistically with TNF-α in inducing CCL20 (MIP-3α) production by NPDF. Thus, the involvement of Th17 cells in the pathophysiology of nasal polyps is emphasised [102].

### 4.12. Thymic Stromal Lymphopoietin (TSLP)

Thymic stromal lymphopoietin (TSLP) is an epithelial cell-derived cytokine implicated in the initiation and persistence of inflammatory pathways. It is released predominantly by ciliated epithelial cells, mast cells, macrophages, and endothelial cells [103,104]. NPDF can secrete TSLP after synergistic stimulation with TNF-α and Th2 cytokines (IL-4 and IL-13). On the other hand, stimulation with Th1 cytokines, such as IFN-γ, can inhibit TSLP production, indicating its regulatory role in dendritic cell activation [105,106]. Furthermore, NPDF can produce TSLP after the activation of TLR2 by fungi such as Alternaria, which could also facilitate the development and exacerbation of type 2 nasal inflammation in patients with CRSwNP [107]. Consequently, the production of TSLP by fibroblasts suggests that these cells may play a role in the development and regulation of Th2 inflammation [108].

### 4.13. Overall Effects of Cytokines Produced by NPDF in CRSwNP

**Figure 3:**
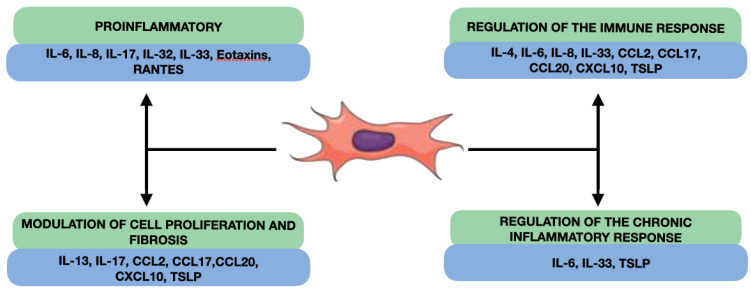
Overall effects of cytokines produced by NDPF.

Our review reveals that fibroblast-produced interleukins in nasal polyps can have several effects on the inflammation and pathogenesis of CRSwNP, such as the promotion of inflammation, the regulation of the immune response, the modulation of cell proliferation and fibrosis, and the participation in the regulation of the chronic inflammatory response (Figure 3). NPDF can secrete proinflammatory interleukins, such as IL-6 and IL-8, which can act as proinflammatory mediators in the nasal cavity [109]. Furthermore, NPDF-produced interleukins can also interact with the cells of the local immune system and inflammatory cells and modulate the immune response in nasal polyps (Figure 2). Some interleukins produced by fibroblasts, such as TGF-β, and IL-4, or platelet-derived growth factor (PDGF) may have profibrogenic effects, stimulating cell proliferation and ECM production, such as collagen and proteoglycans [110,111]. This may contribute to the formation of fibrous tissue characteristic of nasal polyps and to the remodelling of connective tissue. Finally, the NPDF-produced interleukins can also be involved in the regulation of the chronic inflammatory response in nasal polyps, interacting with other cells and inflammatory mediators. For example, IL-6 may act as a proinflammatory and proangiogenic mediator in nasal polyps, contributing to chronic inflammation and polyp growth [112].

**Figure 4:**
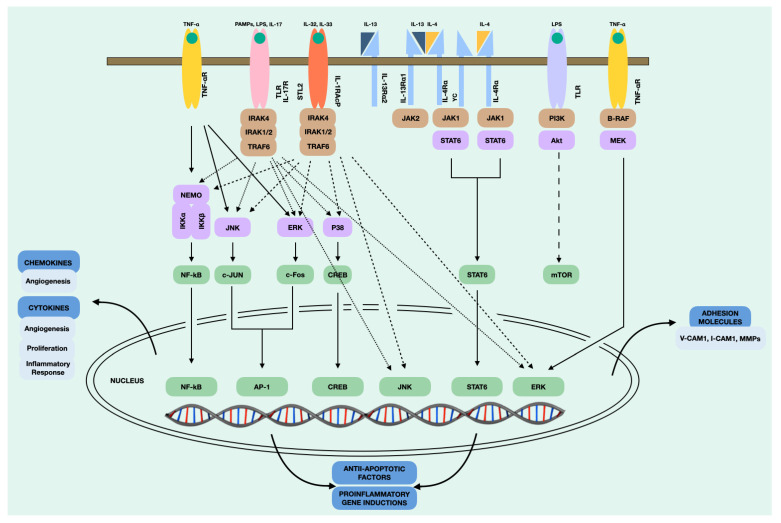
Intracellular signalling pathways of the NDPF.

In addition, NPDF, upon stimulation, trigger intracellular signals that lead to the subsequent secretion of cytokines. This process involves a series of biochemical and molecular events that allow the transmission of the signal through the cell, eventually leading to a specific cellular response. It is important to note that intracellular signalling is a highly regulated and complex process that can vary depending on the cell type, the type of signal, and the cellular context. In our case, we found numerous intracellular signalling pathways in the NPDF after stimulation. The main intracellular signalling pathways that take place in the NPDF are shown in Figure 4. The NF-kB pathway is involved in the induction of inflammatory cytokines, such as RANTES, and the activation of chronic inflammation [113]. The JAK1/STAT6 pathway is stimulated primarily by IL-4 and IL-13, which produce the Th2 immune response and macrophage activation [114]. On the other hand, MAPK/ERK is a complex pathway that participates in the proliferation and cell cycle [115], which, in the case of NPDF, appears to be activated by TNF-α for CCL2 production. Recent studies such as that of Nemec et al. [116] demonstrated that a structurally guided chemical–genetic approach could be rationally applied to target kinase receptors. Therefore, we believe that further targeted studies are needed on these intracellular signalling pathways that may be susceptible to inhibition.

## 5. Limitations

This review has a few limitations. However, we believe that due to the search criteria used, since the articles included only evaluated cytokine production after fibroblast stimulation, we cannot verify the inhibitory role that certain substances can play in fibroblast activity, as well as their constitutive production without prior stimulation, as described by Homma et al. [69]. Additionally, the production of other cytokines not included in the review may have been described in articles prior to 2000 excluded from our study.

## 6. Future Directions

Currently, therapeutic targets in CRSwNP focus on various interleukins such as IL-4, IL-5, and IL-13 produced by Th2 lymphocytes or immunoglobulins such as IgE, produced by B lymphocytes or plasma cells [117,118,119]. The purpose of this review was to evaluate the inflammatory role of NPDF as a major source of cytokine production in the matrix of nasal polyps. Indeed, NPDF produces a great diversity of cytokines that act by activating different inflammatory cells, being directly or indirectly involved in all three inflammatory endotypes T1, T2, and T3. Therefore, we believe that it is interesting to evaluate the role of NPDF as a possible therapeutic target. Future research is needed to determine whether fibroblast inhibition can block nasal polyp production or produce less aggressive CRSwNP.

In addition, we believe it is of interest to evaluate the different behaviours of the fibroblast within other locations of the nasal cavity such as the floor of the nasal fossa, where nasal polyps do not develop [120,121].

## Figures and Tables

**Table 1:** Detailed criteria for inclusion/exclusion of studies.

Criteria	Inclusion Criteria	Exclusion Criteria
Population	Patients with chronic rhinosinusitis with nasal polyposis (CRSwNP)	All other diseases
Intervention/Comparators	Stimulation of nasal-polyp-derived fibroblasts (NPDF)	Inhibition of NPDF Use of drugs on NPDF
Outcomes	Cytokines and/or chemokines	Other type of proteins Inhibition of a cytokine or inflammatory pathway
Study Design	Any study type (clinical trials, prospective and retrospective studies, in vitro or ex vivo) Articles published 1 January 2000 to 1 May 2022	Case reports and animal studies Articles published before 1 January 2000
Language	Articles in English ^a^	All non-English articles

^a^ Citation retrieval was not limited by language. Records were categorized based on language during the title-and-abstract screening stage, and non-English abstracts were excluded. English abstracts with non-English articles were excluded at the full-text screening stage. Abbreviations: CRSwNP, chronic rhinosinusitis with nasal polyposis; NPDF, nasal-polyp-derived fibroblasts.

**Table 2:** Summary of studies investigating cytokines produced by NPDF.

Author (Year)	Aim	Features of Patients	Previous Treatment	Control Group Fibroblasts	Stimulation by	ARN Detection	Immunoassay	Cytokine Produced	Outcome
Saji (2000)	To assess the production of RANTES cytokine by NPDF	No other associated disease	No comment	No	TNF-⍺ IL-1β	RT-PCR	ELISA	CCL5 (RANTES)	RANTES from NPDF was expressed after stimulation with TNF-⍺ and IL-1β
Yamada (2001)	To assess if Syk is involved in the RANTES production	No comment	No comment	Nasal polyps	TNF-⍺ IL-1β	No	Western Blot	CCL5 (RANTES)	TNF-⍺ induced RANTES production in an independent manner at the Syk-expression level
Liu (2002)	To evaluate the production of IL-6 and COX-2 by NPDF	No history of nasal allergy, asthma, or aspirin sensitivity	Not regular topical or oral medication within 3 weeks	Inferior turbinate mucosa	IL-1⍺ TNF-⍺	Northern Blot in situ hybridization	No	IL-6	IL-6 and COX-2 were expressed with a stimulatory effect of IL-1⍺ and TNF-⍺
Olsson (2003)	Proinflammatory functions of NPDF after PBMC mediators	No comment	Not anti-inflammatory treatment 2 weeks before	No	Dp-stimulated PBMC supernatants	No	Flow cytometry ELISA	Il-6 IL-8	IL-6 and IL-8 were significantly higher in NPDFs cultured with Dp-stimulated PBMC supernatants compared to nonstimulated supernatants
Yoshikawa (2003)	To assess the expression IL-13Rs	No comment	No comment	Lung and skin	TNF-⍺ IL-4	RT-PCR	Flow cytometry ELISA	IL-13R⍺2	TNF-⍺ and IL-4 upregulated the cell surface expression of IL-13R⍺2 in NPDF
Steinke (2004)	To assess the effect of IL-4 and IL-13 on CysLT receptor expression on NPDF	No comment	No oral steroids or leukotriene modifier within 4 weeks	No	IL-4 IL-13	RT-PCR	Flow cytometry	IL-6 CCL11 (eotaxin-1) TFG-β1 TFG-β2	IL-4 induced changes in the mRNA and protein expression of fibrotic (TFG-β1, TFG-β2) and inflammatory cytokines and chemokines (IL-6 and CCL11) by NPDF
Nonaka (2004)	To assess eotaxin production by NPDF	No comment	No comment	Uncinate process mucosa, lung and skin	IL-4 IL-13 TGF-β1 LPS	RT-PCR	ELISA	Eotaxin	LPS was necessary for IL-4 to strongly induce eotaxin in NPDF
Shun (2005)	To assess CCL2 expression on NPDF	No history of nasal allergy, asthma, or aspirin sensitivity	Not regular topical or oral medication within 3 weeks	Inferior turbinate mucosa	TNF-⍺	Northern Blot	Inmunohistochemistry	CCL2 (MCP-1)	CCL2 synthesis by NPDF may promote the macrophage recruitment
Yoshifuku (2007)	To assess the expression of RANTES and eotaxin in NPDF	(1) Eosinophilic group >100 per field (2) Noneosinophilic group ≤10 per field	No treatment administered at least 2 weeks prior to surgery	Unstimulated cells of both groups	TNF-⍺ IL-4	RT-PCR	ELISA	CCL5 (RANTES) eotaxin	Eotaxin had an important role in the selective recruitment of eosinophils in the eosinophilic group.
Nonaka (2007)	To assess the production of MCP-4 by NPDF	No associated airway disease	No comment	No	IL-4 TLRs	RT-PCR	ELISA	CCL13 (MCP-4)	The induction of CCL13 by TLRs and IL-4 suggested an important role for NPDF in regulating eosinophilic infiltration
Lin (2007)	To assess the CCL2 expression on NPDF	No history of nasal allergy, asthma, or aspirin sensitivity	No regular topical or oral medication within 3 weeks	Inferior turbinate mucosa	TNF-⍺	Northern Blot	Western Blot	CCL2 (MCP-1)	TNF-α contributed to NP through the induction of CCL2 expression in NPDF
Fukumoto (2008)	To assess if NPDF produced TARC	No comment	No comment	Uncinate process mucosa and lung	IL-4 LPS	RT-PCR	ELISA	CCL17 (TARC)	A combined stimulation with LPS and IL-4 induced CCL17 in NPDF
Nonaka (2008)	To assess if NPDF produced TARC	Allergic chronic sinusitis but no associated lower airway disease	No comment	No	IL-4 TLRs	RT-PCR	ELISA	CCL17 (TARC)	A combined stimulation with TLR ligands and IL-4 induced TARC in NPDF
Nonaka (2008)	To assess the expression of TARC by NPDF	Allergic chronic sinusitis	No comment	No	TNF-⍺ IL-4 poly I:C	RT-PCR	ELISA	CCL17 (TARC)	A combined stimulation with poly I:C, IL-4, and TNF-⍺ resulted in substantial amounts of TARC in NPDF
Nonaka (2009)	To assess the expression of CCL20 by NPDF	Two atopic patients	No comment	No	TNF-α IL-4 IL-17	RT-PCR	ELISA	CCL20 (MIP-3α)	IL-17A and IL-17E contributed to the infiltration of Th17 cells and DC through the production of CCL20 by NPDF
Nonaka (2010)	To assess if NPDF produced CCL20	3 atopic patients, 2 of which with asthma	No comment	No	IL-1β TNF-α TLRs	RT-PCR	ELISA	CCL20 (MIP-3α)	NPDF recruiting DCs via the TLR/proinflammatory cytokine induced the production of CCL20
Nonaka (2010)	To assess the expression of TSLP on NPDF	No comment	No comment	No	IL-4 TNF-α IFN-ɣ IL-10 IL-13	RT-PCR	ELISA	TSLP	TNF-α and Th2 cytokine (IL-4 or IL-13) synergistically induced TSLP for the development and regulation of Th2 cells
Shun (2011)	Hypoxia activates NPDF which induce Cyr61, VEGF, and IL-8	No history of nasal allergy, asthma, or aspirin sensitivity	No regular topical or oral medication within 3 weeks	No	Cyr61	No	Inmunohistochemistry Western Blot ELISA	IL-8	Under hypoxia, NPDF may induce VEGF and IL-8
Yoshikawa (2013)	To assess the expression of CXCL10	3 different groups of CRS patients: (1) NP without asthma; (2) NP and aspirin-tolerant asthma (ATA); (3) NP and aspirin-intolerant asthma (AIA).	No comment	Middle turbinate mucosa and middle meatus mucosa	TNF-α IFN-ɣ IFN-β poly I:C	qRT-PCR	ELISA	CXCL10	The ATA and AIA groups showed a significantly enhanced expression of CXCL 10 after poly IC
Homma (2013)	To assess the level of cytokine and chemokine release from the NPDF and its effect on IL-17A	(1) Eosinophilic group >100 per field (2) Noneosinophilic group <100 per field	No systemic or topical corticosteroids or other immune-modulating drugs for at least 1 month before	Sphenoid mucosa	IL-17A	qRT-PCR	Cytokine assay system	IL-6 IL-8 IL-9 CCL2 (MCP-1) G-CSF	NPDF showed an enhanced basal secretion of MCP-1 and IL-17A stimulated secretion of IL-6, IL-9, and G-CSF.
Niu (2014)	To assess the expression of IL-8 and CXCL1 by NPDF	No comment	Not regular topical or oral medication at least 1 month before	No	IL-17	RT-PCR	No	CXCL1 IL-8	NPDF produced CXCL1 and IL-8 which play a role in neutrophil recruitment
Cho (2014)	To determine PGE2 effect on the levels of IL-6 and IL-8	No comment	No comment	No	PGE2	RT-PCR	Western blot ELISA	IL-6 IL-8	PGE2 increased IL-6 expression via the EP2 and EP4 receptors and IL-8 expression via the EP4 receptor in NPDF
Cho (2014)	To assess proinflammatory cytokines and MMPs	No history of allergy, asthma, or aspirin sensitivity	No oral antiallergic agents for at least 2 months	No	LPS	Microarray RT-PCR	Western blot ELISA	IL-6 IL-8	LPS induced the production and expression of IL-6, IL-8, and MMPs in NPDF
Shin (2016)	To assess the role of PPRs and TLRs in the production of cytokines by NPDF stimulated with airborne fungi	No history of allergy or asthma	No oral or topical medications at least 4 weeks before	No	Alternaria and Aspergillus	RT-PCR	ELISA	IL-6 IL-8	Alternaria induced the production of IL-6 and IL-8 from NPDF, through the interaction of TLRs
Shin (2016)	To assess if airborne fungi activate NPDF to produce TSLP	No history of allergy or asthma	No oral or topical medications at least 4 weeks before	No	Alternaria and Aspergillus	RT-PCR	No	TSLP	Fungi induced TSLP production by NPDF
Cho (2016)	To investigate the mechanism of IL-32 expression induced by LPS in NPDF	No history of allergy, asthma, or aspirin sensitivity	No oral or topical medications during the previous 8 weeks before	Inferior turbinate mucosa	TNF-α IFN-ɣ TGF-β1 IL-1β LPS	RT-PCR	Western blot	IL-32	LPS induced IL-32 expression in NPDF through the TLR4-JNK-AKT-CREB signalling pathway
Shimizu (2017)	To assess if coagulation factors can produce the release of cytokines and extracellular matrix proteins from NPDF	No comment	No comment	No	Thrombin FXa PAR agonist peptides	RT-PCR	Inmunoassay kit	TGF-β1 CCL11(eotaxin-1) IL-6 IL-8	Coagulation factors induced the release of cytokines and ECM protein by NPDF
Imoto (2022)	To assess if leptin release is related to severity of eosinophilic CRSwNP	Eosinophilic CRSwNP	No oral steroids intake for at least 4 weeks before	No CRS	Leptin IL-4 IL-13	RT-PCR	Western blot ELISA	CCL26 (eotaxin-3)	Leptin significantly augmented eotaxin-3 expression in NPDF
Cheng (2022)	To assess the role of IL-17 in pathogenesis od CRSwNP	No comment	No nasal decongestants or antihistamines were administered for 4 weeks before	Untreated fibroblasts	TNF-α	RT-PCR	Western blot ELISA	IL-17A	TNF-α increased levels of IL-17A in NPDF through the PI3K/Akt/HIF-1 α pathway
Lee (2022)	To assess the inflammatory effects of PM10 in NPDF	No comment	No oral or nasal corticosteroids or antibiotics for 4 weeks before	No	PM10	RT-PCR	Western blot ELISA	IL-4 IL-6 IL-33 CXCL1	Increased expression of IL-6, IL-4, IL-33, and CXCL1 on PM10-treated human NPDF

## Data Availability

The study did not report any data.
